# Current Concentrations of Zn, Cu, and As in Piggery Wastewater Compromise Nutrient Removals in Microalgae–Bacteria Photobioreactors Due to Altered Microbial Communities

**DOI:** 10.3390/biology11081176

**Published:** 2022-08-05

**Authors:** Javiera Collao, Pedro Antonio García-Encina, Saúl Blanco, Silvia Bolado-Rodríguez, Nuria Fernandez-Gonzalez

**Affiliations:** 1 Department of Chemical Engineering and Environmental Technology, University of Valladolid, Dr. Mergelina s/n, 47011 Valladolid, Spain; 2 Institute of Sustainable Processes (ISP), University of Valladolid, Dr. Mergelina s/n, 47011 Valladolid, Spain; 3 Department of Biodiversity and Environmental Management, University of León, 24071 León, Spain; 4 Department of Systems Biology, Spanish Center for Biotechnology, CSIC, C/Darwin n°3, 28049 Madrid, Spain

**Keywords:** heavy metals, microbiome, nutrient removal, swine, toxic elements

## Abstract

**Simple Summary:**

Photobioreactor systems based on consortia of microalgae and bacteria are a promising, efficient and sustainable alternative for treatment of wastewaters with high nitrogen content, such as piggery wastewater. In these biological systems, microorganisms play a key role in wastewater treatment by degradation of organic matter and accumulation of nutrients into the generated biomass. However, these wastewaters often contain high concentrations of zinc, copper and arsenic, which can severely affect the activity and growth of microorganisms, and so, the wastewater treatment performance. This article studies the effect of high concentrations of zinc, copper and arsenic on microbial communities, specifically microalgae and bacteria, in photobioreactors treating piggery wastewater, with the aim of elucidating their impact on wastewater treatment performance. For this purpose, the growth of microalgae and the composition and structure of bacterial communities exposed to these pollutants were studied. The performance of the reactors was also evaluated by determining the removal of nutrients, zinc, copper and arsenic. The results showed that high concentrations of zinc, copper and arsenic in piggery wastewater significantly affect the microbiome of the reactors without recovery after exposure to these contaminants, resulting in poorer performance of the reactors and compromising the environmental and health impact of treated effluents.

**Abstract:**

The treatment of pig manure is a major environmental issue, and photobioreactors containing consortia of microalgae and bacteria have proven to be a promising and sustainable treatment alternative. This work studies the effect of Cu, Zn and As, three toxic elements frequently present in piggery wastewater, on the performance and microbiome of photobioreactors. After dopage with Zn (100 mg/L), Cu (100 mg/L), and As (500 µg/L), the high biomass uptake of Zn (69–81%) and Cu (81–83%) decreased the carbon removal in the photobioreactors, inhibited the growth of *Chlorella* sp., and affected heterotrophic bacterial populations. The biomass As uptake result was low (19%) and actually promoted microalgae growth. The presence of Cu and As decreased nitrogen removal, reducing the abundance of denitrifying bacterial populations. The results showed that metal(loid)s significantly affected 24 bacterial genera and that they did not recover after exposure. Therefore, this study makes an important contribution on the impact of the presence of metal(loid)s in piggery wastewater that compromises the overall performance of PBRs, and so, the environmental and health impact of treated effluents.

## 1. Introduction

The large increase in pork demand has promoted intensive pig farming, producing large amounts of piggery wastewater (PWW) with high levels of nutrients, such as carbon, nitrogen (particularly ammonia), and phosphorus. These nutrients can cause serious water eutrophication problems when untreated PWW is discharged into the environment [1,2]. In addition, various pollutants, such as heavy metals (HMs), are present in PWW. Zinc (Zn) and copper (Cu) are essential components for pigs’ growth and often used as feed additives to promote growth, but animals only absorb a small percentage (10–20%) of these metals and the rest is excreted through urine and feces and accumulated in PWW. Consequently, high concentrations of Zn and Cu can be found in PWW, ranging from 12 to 234 mg/L of Zn and 4.7 to 148 mg/L of Cu [3,4]. In addition, other toxic elements, such as arsenic (As), are frequently found in the well water used on the farms in regions where this metalloid is present in the rock matrices of aquifers, such as in the Cenozoic Duero basin in Spain [5], where this study was conducted. Arsenic has been found in PWW, in some cases achieving concentrations as high as 690 μg/L [6]. After discharge, these non-degradable pollutants accumulate in rivers, lakes, seas, and soils, entering food chains and causing serious hazards to the environment and human health. Therefore, it is necessary to remove these contaminants from PWW before discharge.

Microalgae–bacteria photobioreactors (PBRs) for wastewater treatment have been extensively studied over the past two decades because they are promising systems to remove organic matter, inorganic compounds, and contaminants at minimal energy cost [7]. PBRs are based on a beneficial relationship between microalgae and bacteria. Microalgae use sunlight, nutrients and carbon dioxide produced during the degradation of organic matter by heterotrophic bacteria. In turn, oxygen released by microalgae during photosynthesis is used by heterotrophic bacteria to oxidize organic matter and grow [8]. PBRs have been proven as efficient and environmental friendly treatments for wastewaters with high nutrient loads, such as PWW [9]. In addition, some studies have reported that microalgae–bacteria PBRs could be an alternative for bioremediation of PWW contaminated with HMs and As [6,10,11].

However, previous studies on bioremediation of metals from wastewater have been carried out by investigating microalgae and bacteria separately, or by using synthetic media or PWW with low concentrations of toxic elements. Until now, few studies report the removal of PWW with high metal(loid) concentrations using microalgae–bacteria PBRs systems. In this context, many species of microalgae have the ability to growth and remove high concentrations of metal(loid)s through detoxification mechanisms from synthetic solutions [12,13]. Cultures of *Chlorella*, a microalgae frequently used for wastewater treatment, achieved removals of 46% from a solution with around 200 mg/L of Zn, 30.37% from a solution containing about 60 mg/L of Cu [13] and 66% of As from synthetic waste with 500 mg/L of As [14]. Moreover, it has been documented that different bacteria, such as *Pseudomonas*, *Micrococcus*, *Enterobacter* and *Bacillus* sp. (belonging to the *Bacteroidetes*, *Proteobacteria*, *Actinobacteria* and *Firmicutes* phyla, respectively) tolerate and remove high concentrations of HMs and As from different environments due to their high multi-metal resistance [15].

Comparatively, the use of microalgae and bacteria co-cultures could improve the elimination of these toxins. Previous studies have demonstrated that bacteria can mitigate metal toxicity and enhance removal through detoxification mechanisms in microalgae co-culture [16,17]. For example, the bacteria *Alteromonas macleodii* promote the excretion of As from *Dunaliella salina* when working in a synthetic medium with 1.112 mg/L of As in the presence of 0.112 mg/L of phosphorus [17]. Likewise, higher removals of Zn, Cu and As were found during co-culture of activated sludge and a microalgae, either *Chlorella vulgaris* or *Scenedesmus obliquus*, than during microalgae monoculture [6].

To date, the few studies on metal removal using wastewater treatment PBRs have paid attention to the effect of toxins on microalgae growth, overlooking their influence on bacterial communities and even less on nutrient removal. In this context, the effect of toxic elements on microbial populations can be decisive for reactor stability and nutrient removals [18,19]. Previous studies with axenic microalgae cultures and with activated sludges have shown a remarkable effect of toxic elements on both microalgae and bacterial communities. For example, arsenic is a toxic metalloid and can be detrimental to basic cell function when arsenate anion replaces phosphate in cellular metabolic processes [20]. It has been demonstrated that high Zn, Cu, and As concentrations can replace metal atoms in the active sites of certain enzymes and decrease the growth of microalgae by inhibiting chlorophyll [19,21,22]. Furthermore, bacterial diversity and performance of biological wastewater treatment systems decreased in the presence of Zn [23] and Cu [24]. In addition, HMs have shown the resilience of some nitrifying and denitrifying bacteria, which are able to adapt to the Cu toxicity and become the dominant species [24]. These studies show the importance of studying the possible impact of treating wastewater with high concentrations of HMs and As on microalgae and bacterial communities in PBRs due to their influence on nutrient removal and reactor performance.

To our knowledge, this study is the first comprehensive investigation with the objective of evaluating the effect of high concentrations of HMs (Zn and Cu) and As on performance and microbial communities in microalgae–bacterial PBRs during wastewater treatment. For this purpose, 3 L lab-scale PBRs inoculated with a microalgae culture and fed with PWW, working in continuous mode at hydraulic retention times of 5 days, were doped with 100 mg/L of Zn, 100 mg/L of Cu, and 500 μg/L of As. These concentrations were chosen based on the highest values frequently reported worldwide for these contaminants in PWW to analyze the most extreme treatment scenarios [3,4,6]. The wastewater treatment performance, growth of microalgae, and diversity and structure of bacterial communities were analyzed before, during, and after exposure of PBRs to HMs and As. The article clearly showed that high concentrations of metal(loid)s in PWW reduce the quality of treated effluents due to a significant effect on the microbial communities of the PBRs. The increased removal of Zn and Cu, by biomass absorption, caused inhibition of *Chlorella* sp. Growth and significant changes in bacterial communities, resulting in reduced carbon removal. Despite its greater toxicity, As had less impact on the reactor microbiome than Zn and Cu, but lower removals of this metalloid were achieved, contributing to the generation of contaminated effluent.

## 2. Materials and Methods

### 2.1. Piggery Wastewater and Inoculum

Two batches of raw PWW were collected just before the start of each experiment (A and B) from a farm in Segovia (Spain) at different times and stored at 4 °C until the end of the experiment. The raw PWW was centrifuged at 10,000 rpm for 10 min and the supernatant was diluted at 5% *v/v* with tap water. This diluted wastewater was used as the reactor feed. The dilution percentage was selected to avoid microbial inhibition as a consequence of PWW toxicity, mainly due to high ammonium concentrations [10,25]. The feed composition for each set of experiments is shown in Table 1. The microalgae culture used as the inoculum (with >95% of *Chlorella* sp.) was obtained from an indoor open reactor treating PWW located at the Institute of Sustainable Processes (University of Valladolid, Spain, 41.655951 N 4.716837 W).

### 2.2. Photobioreactors, Operational Conditions, and Experimental Design

To observe the effect of HMs and As on the performance of the PBRs, two sets of experiments (A and B) were performed independently, applying identical operating conditions and methodology but using a different batch of raw PWW as feed. For both series of experiments, four open PBRs (R1, R2, R3, and R4) with a capacity of 3 L (16 cm depth) each were used. The PBRs were operated under indoor conditions and illuminated at 1400 μmol/m^2^/s by LED lamps, applying light/dark cycles of 12:12 h:h (08:00 h to 20:00 h). The room temperature was controlled to maintain a temperature in the PBRs in the range of 28 to 33 °C, which is considered to be in the optimal range for microalgae growth [25]. The PBRs were equipped with internal water pumps to mix the microalgal–bacterial culture broth and pH electrodes. The pH was maintained at 8 through the automatic addition of pure CO_2_. The PBRs were inoculated to 10% of their capacity with a microalgae culture and filled to the final working volume (3 L) with PWW diluted at 5% *v/v* with tap water. The feed was pumped to the PBRs by a multi-channel self-control pump (Watson-Marlow, Falmouth, UK). The volume fluctuations were always maintained below the 20% PBRs’ volume. For this, tap water was added to compensate for evaporation, whenever necessary. The PBRs were operated in continuous mode at a hydraulic retention time (HRT) of 5 days. The HRT and operation conditions were chosen according to typical values reported for wastewater treatment in microalgae–bacteria systems [9].

When the values of the nutrient concentrations in the effluent and the growth of the microalgae became constant over time, i.e., steady state, the microalgae–bacteria PBRs were mixed to start the experiments with homogeneous microbiomes. This event was considered day 0. The experiments were divided in three phases. First, after achieving steady state of the PBRs and mixing of the cultures, the four PBRs continued to be fed with 5% PWW for 7 days (phase I). Then, from day 7 to day 29, R2, R3, and R4 were fed with PWW diluted at 5% and doped with 100 mg/L of Zn, 100 mg/L of Cu, and 500 μg/L of As, respectively (phase II). These concentrations were chosen according to the highest values reported in the literature in PWW for these elements [3,6]. It should be noted that the concentrations of Zn, Cu, and As in the 5% PWW used as feed (Table 1) were very low. Therefore, their influence on the final concentration of the doped feed was negligible. The doped feeds were prepared at nominal concentrations of Zn, Cu, and As using CuCl_2_·2H_2_O, ZnCl_2_, or Na_2_HAsO_4_·7H_2_O (Sigma Aldrich) and kept at 4 °C. From day 29 to day 36, the four PBRs were again fed with PWW diluted at 5% without the addition of Zn, Cu, or As (phase III). The feed of R1, which was used as a control, was not doped with Zn, Cu, or As in any phase of the experiments.

The temperature (T) and dissolved oxygen concentration (DO) of the PBRs were measured at least daily using an oximeter (OXI 330i, WTW, Germany). The pH was continuously monitored and controlled using an electrode (Crison pH 5330) connected to a Crison multimeter M44 (Crison instrument) and pH electrodes were calibrated every two days. In addition, the cumulative inlet and outlet flow rates of each reactor were determined on a daily basis to calculate the removal efficiencies. The maximum quantum yield of photosystem II (PSII), expressed as the ratio of variable to maximum fluorescence (F_v_/F_m_) was measured every two days to determine the physiological state of microalgae cells using an AquaPen-C AP110-C fluorometer (Photon Systems Instruments, Czech Republic), following the manufacturer’s instructions. F_v_/F_m_ is widely used to evaluate the damage of photosynthetic activity of microalgae exposed to toxic contaminants or stress conditions [26]. The chemical parameters were measured every two days in feed and effluent samples collected at the inlet and outlet of each reactor. A fraction of the sample was used to determine the total suspended solid (TSS) concentrations by standard methods [27]. Another fraction was used for chemical composition analysis. In the case of the effluent, the sample was centrifuged at 10,000 rpm for 5 min to obtain the liquid phase. Total organic carbon (TOC) and total nitrogen (TN) were measured using a TOC-V CSH analyzer equipped with a TNM-1 chemiluminescence module (Shimadzu, Kyoto, Japan). The ammonia nitrogen (NH_4_^+^-N) concentration was determined using an ammonium selective electrode (Orion 9512 HPWBNWP ammonia, Thermo Scientific, Waltham, MA, USA) and total phosphorus (TP) according to standard methods. To analyze the concentrations of Zn, Cu, and As, aliquots of HNO_3_ (0.1 M) were added to the feed and the liquid phase of the effluent before being stored at 4 °C until analysis. The extraction of HMs and As from the acidified samples was carried out by acid digestion in a microwave oven (Ethos Sel and Ultrawave, Milestone), according to internal analytical standard methods of the Instrumental Techniques Laboratory (LTI-UVa). The concentration of Cu and Zn was determined by inductively coupled plasma spectrometry together with an optical emission spectrophotometer (ICP-OES) (Varian 725-ES, Agilent, Santa Clara, CA, USA) and As was measured using an inductively coupled plasma source mass spectrometer (ICP-MS) in an octopolar reaction system (HP 7500c, Agilent, USA). All plastic and glass containers were washed in diluted HNO_3_ (10% *v*/*v*) for 24 h and rinsed 3 times with Milli-Q water before and after use with HMs and As [28].

The removal efficiencies (RE) of TOC, TN, NH_4_^+^-N, TP, Zn, Cu and As were calculated according to Equation (1) [10] and the uptake of Zn, Cu, and As by the biomass (q) was calculated according to Equation (2) [28]:

(1) $$\mathrm{RE}\;\left(\%\right)=\frac{\left(C_{\mathrm{input}}\cdot {\;Q}_{\mathrm{input}}-{\;C}_{\mathrm{output}}\cdot \;\;Q_{\mathrm{output}}\right)}{C_{\mathrm{input}}\cdot \;Q_{\mathrm{input}}}\times 100$$

(2) $$q\;\left(\frac{\mathrm{mg}}{g}\right)=\frac{\left(C_{\mathrm{input}}\cdot {\;Q}_{\mathrm{input}}-{\;C}_{\mathrm{output}}\cdot \;\;Q_{\mathrm{output}}\right)}{m\cdot {\;Q}_{\mathrm{output}}}$$

where C_input_ and C_output_ (mg/L) represent the concentrations of TOC, TN, NH_4_^+^-N, TP, Zn, Cu, and As in the feed and liquid phase of the effluent of the PBRs, respectively. The Q_input_ and Q_output_ (L/d) correspond to the feed and effluent flow of the PBRs, and m (g/L) is the effluent suspended solids concentration.

To identify and quantify the microalgal communities, culture samples were periodically taken from inside of PBRs, preserved with 5% lugol and 10% formaldehyde and stored at 4 °C [29], until microscopic observation (Leica DM 4000 B) in a bright field with 100 × magnification. The cell count was performed in triplicate using a Neubauer chamber (Marienfeld, Germany). For the sets of experiment B, biomass samples were collected from inside the PBRs with sterile tubes and stored at −80 °C until DNA extraction for 16S rDNA gene amplification and library construction.

### 2.3. Genomic DNA Extraction and Amplicon Sequencing

The analysis of the bacterial communities was performed in the sets of experiment B, being the sampling times for this analysis selected from the reactor performance results of experiment A. Samples of the microalgae–bacteria culture were collected to study changes in the bacterial communities of each reactor during: phase I, at the initial day and before adding metal(loid)s (days 0 and 5); phase II, to observe the temporal changes just after adding metal(loid)s and during the steady state (days 9, 15, 22, and 29); and phase III, to observe the recovery of bacterial communities after stopping the addition of metal(loid)s (day 36). Genomic DNA extraction was carried out using a FastTM DNA Spin Kit for soil extraction (MP Biomedicals, Thermo Fisher Scientific), according to the manufacturer’s instructions with the following modifications in the sample preparation and cell rupture procedure: 2 mL of microalgae–bacteria culture were added to a lysing matrix E tube and centrifuged at 14,000 rpm for 5 min using a centrifuge 5430 R (Eppendorf); the supernatant was discarded and the pellet was used to extract genomic DNA. In the cell rupture procedure, the time was reduced to 10 s to avoid DNA degradation and a Mini-Beadbeater-16 (Biospec Products) was used for homogenization. The integrity of the DNA extraction was verified by 0.8% (*w*/*v*) agarose gel electrophoresis. DNA concentrations were determined by fluorometry with a Qubit 4 fluorometer (Thermo Fisher Scientific, Waltham, MA, USA). Next, 16S rDNA gene amplicons of the V3 and V4 region were obtained using the primers described in Klindworth et al. (2013) [30], and libraries for sequencing were built using the Metagenomic Sequencing Library Preparation Illumina protocol (Cod. 15044223 Rev. A). Libraries were multiplexed with a Nextera XT Index Kit and sequenced using a 2 × 300pb paired-end run (MiSeq Reagent kit v3) on a MiSeq Sequencer according to the manufacturer’s instructions (Illumina, San Diego, CA, USA). The quality of the obtained sequences was assessed using the prinseq-lite program [31] with the following parameters (min_lenght: 50, trim_qual_right: 30, trim_qual_type: mean, trim_qual_window: 20). Next, the DADA2 pipeline [32] was used for denoising, paired-ends joining, and chimera depletion starting from paired-ends data. The resulting exact amplicon sequence variants (ASVs) were taxonomically affiliated using the Naïve Bayesian classifier and the Silva v.138 database [33] and grouped at the most detailed taxonomic level that in some cases were specie (species vs. samples reads count table), although most were classified at genus and family level. ASVs that were not classified to a more precise taxonomic level (species) were retained with their last taxonomic level classified. These analyses were performed using QIIME2 plugins [34]. Library preparation, sequencing, and initial bioinformatic analysis were performed by the Foundation for Health Promotion and Biomedical Research of the Valencian Community (FISABIO, Valencia, Spain). Raw reads are available at the Sequence Read Archive (SRA) of the NCBI under the accession number PRJNA778742.

### 2.4. Statistical Analysis and Diversity

The effect of Zn, Cu, and As on PBRs performance was studied by applying an analysis of variance (ANOVA) using Statgraphics Centurion XVIII software, where Tukey’s honestly significant difference (HSD) multiple range test (with a confidence level of 95%) was carried out to identify significant differences between PBRs. In addition, all statistical analyses of bacterial communities were performed in R [35] with vegan [36], phyloseq [37], ampvis2 [38], and microbiome [39] packages. All *p*-values were corrected for multiple comparisons by false discovery rate.

After agglomerating the ASVs to the most detailed taxonomic level, the species vs. samples reads count table was used to analyze the PBRs microbiomes. To determine Alpha diversity indices (richness, diversity and evenness) using the microbiome package in R, 58,200 reads were randomly subsampled for each sample to avoid differences in sequencing depth. The observed species were used as an approximation to richness. Diversity was calculated as the inversed Simpson diversity index (1/D) according to Equation (3):

(3) $$\frac{1}{D}=\frac{1}{\sum_{i=1}^{s}p_{i}^{2}}$$

and evenness was measured with the Simpson evenness (E index) (Equation (4)):

(4) $$E=\frac{1}{\sum_{i=1}^{s}p_{i}^{2}}\times \frac{1}{S}$$

where *S* is the number of observed species (richness), and *p_i_* is the proportion of species *i* relative to the total number of species. As diversity increases, 1/D increases, while E ranges from 0 (no uniformity) to 1, which indicates complete evenness or uniformity. To compare alpha diversity indices during the exposition to Zn, Cu, and As, pair-wise Wilcoxon tests were performed using data from phase II.

To analyze microbiome structures and their relationships with the addition of Zn, Cu, and As, two multivariate analyses were performed. Prior to that, abundance data were filtered to remove bacterial species that were not present in more than 0.1% in any sample. In addition, relative abundances were transformed by the Hellinger method. To analyze the relationship between the microbiome structures and the addition of the metal(loid)s, a transformation-based redundancy analysis (tbRDA) was applied (constrained to the Zn, Cu and As data), which were log-transformed. Unconstrained ordination non-metric multidimensional scaling (NMDS) is an indirect gradient analysis approach that produces an ordination based on a distance or dissimilarity matrix. Hence, NMDS of Bray–Curtis dissimilarities was used to visualize microbiome structure among samples. Permutational Analysis of the Variance (PERMANOVA) was then applied to find significant differences between PBRs in the community structure as determined by Bray–Curtis dissimilarities. To identify which bacteria were the major contributors to the reactors’ microbiome differences detected by PERMANOVA, similarity percentage breakdown (SIMPER [40]) procedure was applied using the Bray–Curtis dissimilarity data after removing samples from phase I. Any bacterial specie that did not contribute with at least 1% to the dissimilarity among samples was discarded. SIMPER results were checked with a Kruskal–Wallis statistical test. Prior to visualization into a heatmap, SIMPER results were agglomerated at the genus level. Spearman rank correlations (ρ) were calculated using the table of ASVs grouped at the genus level with several operational variables (F_v_/F_m_ ratio, effluent concentrations of TOC, TN, ammonia, Zn, Cu, and As) considering phases II and III.

## 3. Results

To study the effect of Zn, Cu, and As on the performance and microbiome of PBRs, two sets of experiments, A and B, were conducted independently using the same conditions but different PWW batches as feed. In each set of experiments, four PBRs were used: one reactor served as the control (R1-control) and three PBRs were doped with 100 mg/L of Zn (R2-Zn), 100 mg/L of Cu (R3-Cu), or 500 μg/L of As (R4-As), respectively. The reactors’ operation was divided into three phases depending on the exposition to toxicants: an initial phase without toxic exposure (phase I, days 0–6); an exposure period in which the PWW used as feed for the PBRs were doped with Zn, Cu, or As, except for the R1-control (phase II, days 7–29); and a recovery period in which all PBRs were fed without toxicants (phase III, days 30–36). After the perturbation initiated at the beginning of phase II, both nutrient concentrations and microalgae growth reached stable values, which could be considered as steady state, during days 15–29. Because of that, comparisons of removal efficiencies between PBRs in phase II were conducted only considering compositions in this steady state period.

### 3.1. Photobioreactor Performance and Removal of Zn, Cu, and As

**Figure 1:**
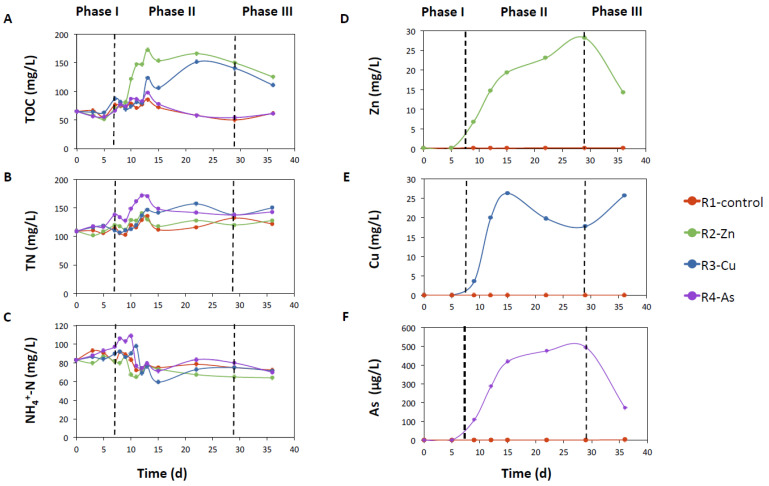
Concentrations of nutrients and Zn, Cu, and As in the effluent for the sets of experiment B in R1-control, R2-Zn, R3-Cu, and R4-As PBRs fed continuously at an HRT of 5 days. TOC (**A**), TN (**B**), NH_4_^+^-N (**C**), Zn (**D**), Cu (**E**), and As (**F**). Vertical dashed lines indicate the change of phase.

Nutrient concentrations in the effluents during phase I were similar between the PBRs in each set of experiments (Figure 1A–C and Figure S1A–C), which resulted in narrow ranges of variation in the removal efficiencies. These ranged between 76–77% for TOC, 48–53% for TN, and 20–24% for NH_4_^+^-N in experiment A, and 91–92% for TOC, 58–61% for TN, and 50–53% for NH_4_^+^-N in experiment B.

The addition of Zn and Cu in phase II notably increased the TOC concentration in the effluent of R2-Zn and R3-Cu PBRs compared to the effluents of R1-control and R4-As (Figure 1A and Figure S1A; *p*-value < 0.05, Table S1). Likewise, a significant reduction in TOC removal efficiency (*p*-value < 0.05, Table S1) was observed in the Zn and Cu doped PBRs compared to R4-As and R1-control (average TOC-RE in experiments A and B on days 15 to 29: R2-Zn: 43 ± 2% and 81 ± 2%, R3-Cu: 56 ± 1% and 83 ± 3%, R4-As: 87 ± 3% and 92 ± 1%, and R1-control: 83 ± 1% and 93 ± 1%). By contrast, the addition of As did not affect the concentrations and removal of TOC (Table S1), and, maintained values close to those observed in R1-control. After toxicants withdrew in phase III, TOC concentrations decreased in the effluents from R2-Zn and R3-Cu, although these values were still higher than TOC concentrations in R1-control and R4-As PBRs. Consequently, TOC removal efficiencies increased in R2-Zn and R3-Cu PBRs during phase III, reaching values of 50% and 85% in R2-Zn and 68% and 86% in R3-Cu on day 36 for experiments A and B, respectively. TOC removal in R4-As remained almost constant after As doping, with values similar to those obtained in phase II and in the R1-control.

The concentration of TN in the effluent was increasing in R3-Cu and R4-As PBRs after the additions of Cu and As (Figure 1B and Figure S1B), with values significantly different compared to the R1-control in both sets of experiments at the end of phase II (*p*-value < 0.05, Table S1). By contrast, the TN concentrations in effluents of R2-Zn and R1-control were similar. Accordingly, the efficiency of TN removals decreased during phase II to averaged values of 38 ± 2% and 46 ± 2% for R3-Cu, and to 42 ± 3% and 47 ± 1% for R4-As for experiments A and B, respectively. In comparison, average TN removal efficiencies of R2-Zn (46 ± 2% and 58 ± 4%, for sets A and B) and R1-control (47 ± 3% and 58 ± 3%) were slightly higher than R3-Cu and R4-As. During phase III, the TN removals altered in phase II partially recovered, reaching values of 49% in R3-Cu (in both experiments) and 52% and 50% in R4-As for experiments A and B, respectively. On the other hand, NH_4_^+^-N effluent concentrations and removal efficiencies of the doped PBRs were not affected by any of the studied elements (Figure 1C and Figure S1C; *p*-value > 0.05, Table S1). The total phosphorus concentration in the effluents remained below the quantification limit (<1 mg/L) throughout the entire operating period.

To calculate the removals and uptakes of Zn, Cu, and As in the PBRs, their concentrations in the doped feed and in the liquid phase of the effluent were determined. The addition of Zn, Cu, and As did not affect the concentration of other metal(loid)s. The concentration of Zn in the effluents of the PBRs not doped with this metal was in the range of 0.2 and 0.6 mg/L in both experiments. In the R1-control, the average Zn removal was 74 ± 9% and 78 ± 0.2% and the average uptake was the same (1.3 ± 0.1 mg/g) for experiments A and B, respectively. The Cu concentration was always below the limit of quantification (<0.1 mg/L) in all the PBRs not doped with this metal. The As concentration in the effluent of the PBRs without As addition varied between a range of 1 and 1.5 μg/L. During phase II, the R1-control reached an average As removal of 39 ± 3% and 51 ± 1% and a low As uptake (1 × 10^−3^ ± 2 × 10^−4^ and 2 × 10^−3^ ± 3 × 10^−3^ mg/g) for experiments A and B, respectively.

In the doped PBRs, Zn, Cu, and As concentrations in the liquid phase of the effluents followed a similar trend in both sets of experiments (Figure 1D–F and Figure S1D–F). The Zn and Cu concentrations of R2 and R3 PBR effluents reached average values of 37 ± 8 and 24 ± 4 mg/L in R2-Zn and 20 ± 1 and 21 ± 4 mg/L in R3-Cu during steady state of phase II for experiments A and B, respectively. Likewise, average removal efficiencies were 69 ± 7% (experiment A) and 81 ± 4% (experiment B) for Zn in R2-Zn and 83 ± 1% (experiment A) and 81 ± 4% (experiment B) for Cu in R3-Cu. These HMs were recovered in the biomass, with uptake capacities of 141 ± 9 and 209 ± 23 mg/g of Zn in R2-Zn and 127 ± 6 and 132 ± 17 mg/g of Cu in R3-Cu for experiments A and B, respectively. During phase III, the Zn concentration in the liquid phase of the R2-Zn effluent decreased remarkably, but the Cu concentration in the R3-Cu effluent remained almost constant even after stopping Cu doping. In contrast to Zn and Cu, a high percentage of the As doped into the feed had left the reactor, leaving in the effluent at the end of phase II, which achieved stable average values of 497 ± 74 μg/L (experiment A) and 462 ± 39 μg/L (experiment B) in R4-As. Uptake in biomass was at mean values of 0.180 ± 0.11 mg/g (experiment A) and 0.208 ± 0.15 mg/g (experiment B) in phase II. In this period, the average As removals were 19 ± 13% for experiment A and 19 ± 11% for experiment B. In phase III the presence of this element in liquid phase of the effluent remarkably decreased once the doping stopped.

### 3.2. Influence of Zn, Cu, and As on Microalgal Growth

**Figure 2:**
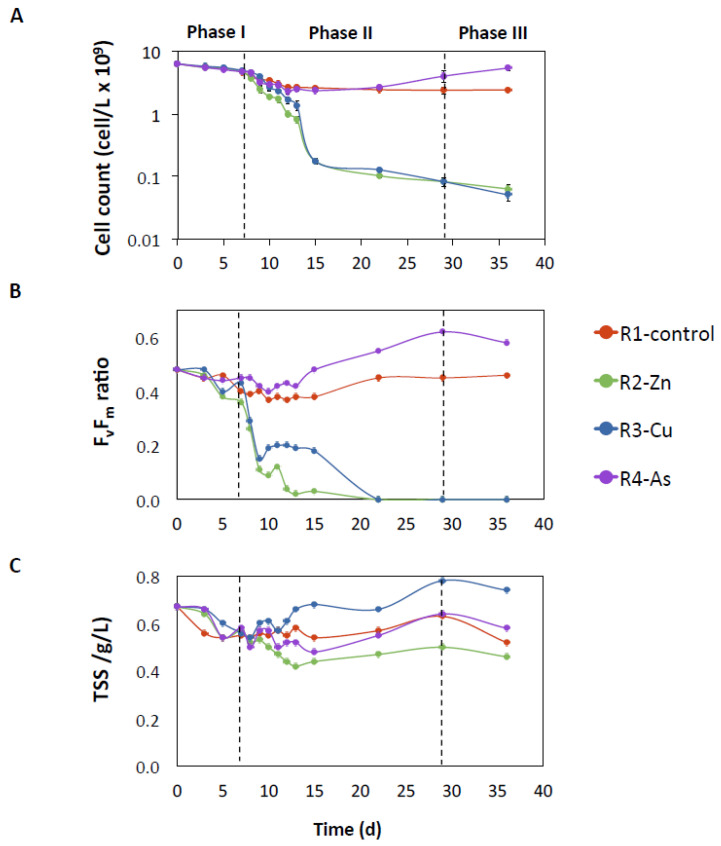
Microalgae growth parameters for the sets of experiment B. Cell count (**A**), maximum quantum yield of PSII by F_v_/F_m_ ratio (**B**), and TSS (**C**) in the R1-control, R2-Zn, R3-Cu, and R4-As PBRs. Horizontal dashed lines indicate the change of operational phase.

*Chlorella* sp. was the only microalgae observed in the PBRs in both sets of experiments. The initial cell concentrations of *Chlorella* sp. in the inoculum of the PBRs (day 0) were in the same range for both sets of experiments (A: 2.72 × 10^9^ cells/L; B: 6.31 × 10^9^ cells/L; Figure 2A and Figure S2A). The addition of Zn and Cu decreased the concentration of *Chlorella* sp. cells in R2-Zn and R3-Cu (phase II) and did not rebound during the recovering phase (phase III). By contrast, the growth of *Chlorella* sp. increased after the addition of As in reactor R4 with respect to the R1-control, reaching values of 1.31 × 10^9^ and 4.04 × 10^9^ cells/L at day 29, and 4.33 × 10^9^ and 5.45 × 10^9^ cells/L at day 36 in the experiments A and B, respectively. The cell concentrations in the R1-control were 0.44 10^9^ and 2.41 × 10^9^ cells/L at day 29 and 0.34 × 10^9^ and 2.44 × 10^9^ cells/L at day 36 in experiments A and B, respectively.

The impacts of Zn, Cu, and As on the physiological state of the microalgae were analyzed by quantifying the maximum quantum yield of photosystem II (F_v_/F_m_ ratio, Figure 2B and Figure S2B). In both experiments, the F_v_/F_m_ ratio followed the same trend as the number of cells, strongly decreasing after the addition of Zn and Cu. The only remarkable difference is that a slower decay was observed in R3-Cu than R2-Zn; however, the F_v_/F_m_ ratios dropped to 0.0 in both PBRs at day 29 without any recovery in phase III. Conversely, the highest F_v_/F_m_ ratio was found in R4-As at the end of phase II (day 29, experiment A: 0.66 and experiment B: 0.62). This value was higher than the maximum F_v_/F_m_ ratio observed in the R1-control (experiment A: 0.38 and experiment B: 0.45).

TSS can reflect biomass growth, although other processes, including physico-chemical transformations, might also affect it. The TSS values measured in R2-Zn and R3-Cu were statistically different than the TSS values of the R1-control (Figure 2C and Figure S2C). In R2-Zn, TSS was lower than TSS in the R1-control with average values of 0.60 and 0.47 TSS g/L in experiments A and B, respectively. By contrast, TSS measurements in R3-Cu were higher than in the R1-control, reaching average values 0.80 and 0.71 TSS g/L in experiments A and B, respectively. Measurements of TSS in R4-As were similar than those observed in the R1-control.

Finally, the DO concentration was determined, given that it was possible indicator of microalgae growth and bacterial activity. In general, a decrease in DO was observed in the first days of exposure to HMs in all doped PBRs (8–12 days of phase II with average values of: R2-Zn: 3.3 ± 0.4, R3-Cu: 2.6 ± 0.4, R4-As: 2.1 ± 0.8 mg/L) compared to the R1-control (average of 4.3 ± 0.2 mg/L). From days 15 to 29 (phase II), the DO concentration in doped PBRs recovered values similar to the R1-control or even slightly higher in the case of R3-Cu (Figure S3, Table S1). When Zn, Cu, and As were removed in phase III, the DO concentrations decreased again in R3-Cu and R4-As.

### 3.3. Bacterial Community Diversity and Structure

Different alpha diversity indices (richness, evenness, and diversity) were used to measure distinct components of bacterial diversity. The indices were calculated in the PBRs biomass and in the raw undiluted PWW used to prepare the feed, which overall had higher diversity and evenness but similar richness than the averaged indices for PBRs (Table S2). The PBRs’ bacterial diversity changed over time. In phase II, with the addition of Zn, Cu, and As, the richness tended to be higher in R2-Zn and lower in R3-Cu (Figure S4). The diversity and evenness indices were also higher in R2-Zn than in the rest of the PBRs, although both diversity and evenness also increased in the R4-As reactor towards the end of phase II. In phase III, richness and diversity decreased in R2-Zn, while diversity and evenness decreased in R4-As. By contrast, diversity and evenness increased in R3-Cu.

**Figure 3:**
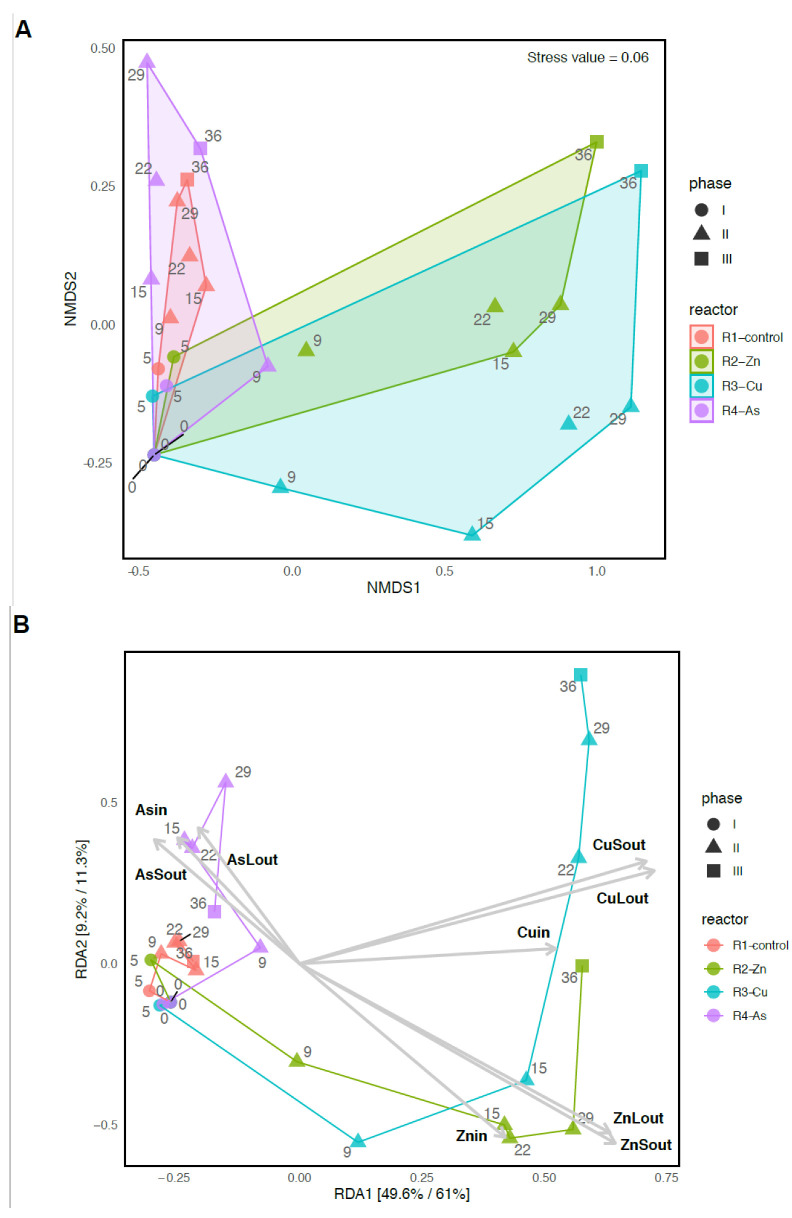
Variation in community composition among PBRs and over time. Non-Metric Multidimensional Scaling Analysis (NMDS) based on the Bray–Curtis distance measure. Prior to the analysis, bacterial species that are not present in more than 0.1% relative abundance in any sample were removed (**A**). Transformed-based redundancy analysis. Total and constrained variance explained by each axis are indicated between brackets. Samples from the same reactor are connected with lines that indicate the temporal change. Operational variables that constrain the ordination are shown with grey vectors. Constrained variables are the concentration of the following: *Znin*—feed Zn, *ZnLout*—effluent liquid phase Zn, *ZnSout*—biomass Zn, *Cuin*—feed Cu, *CuLout*—effluent liquid phase Cu, *CuSout*—biomass Cu, *Asin*—feed As, *AsLout*—effluent liquid phase As, *AsSout*—biomass As (**B**).

The differences in community composition between PBRs over time were determined with Bray–Curtis dissimilarities, visualized in an NMDS ordination (Figure 3A and Figure S5). R2-Zn and R3-Cu species assemblages became very different from the community structures of the R1-control and R4-As PBRs in phase II. These communities did not recover the original structure during phase III. The NMDS analysis also indicated that the structure of the R4-As microbiome was more similar to the R1-control than to the other metal-doped PBRs. This same trend was observed in the structure of the PBRs bacterial communities when analyzed together with the raw PWW sample (Figure S5). A PERMANOVA analysis was used to compare the PBRs’ community assemblages during phase II and assess whether the observed differences after the addition of Zn, Cu, and As were significant. The overall PERMANOVA test indicated that the communities’ compositions were statistically different during the exposition to pollutants (*p*-value < 0.001, Table S3). PERMANOVA pair-wise comparisons (Table S4) showed that among microbiomes, R2-Zn and R3-Cu were statistically different to the R1-control and R4-As (*p*-values < 0.05).

Finally, to analyze the relationship between the microbial community structure and the addition of Zn, Cu, and As, a tbRDA analysis was performed (Figure 3B). In short, in a tbRDA analysis, the variation in bacterial species is regressed against the variables that measure the presence of toxicants. Therefore, variation in bacterial composition was related to metal(loid)s and to a lesser extent to other variables. In the PBRs, the tbRDA analysis indicated that 81.4% of the bacteria variance could be explained by the presence of the toxicants (Table S5).

### 3.4. Temporal Changes on the Bacterial Composition of PBRs’ Microbiome

**Figure 4:**
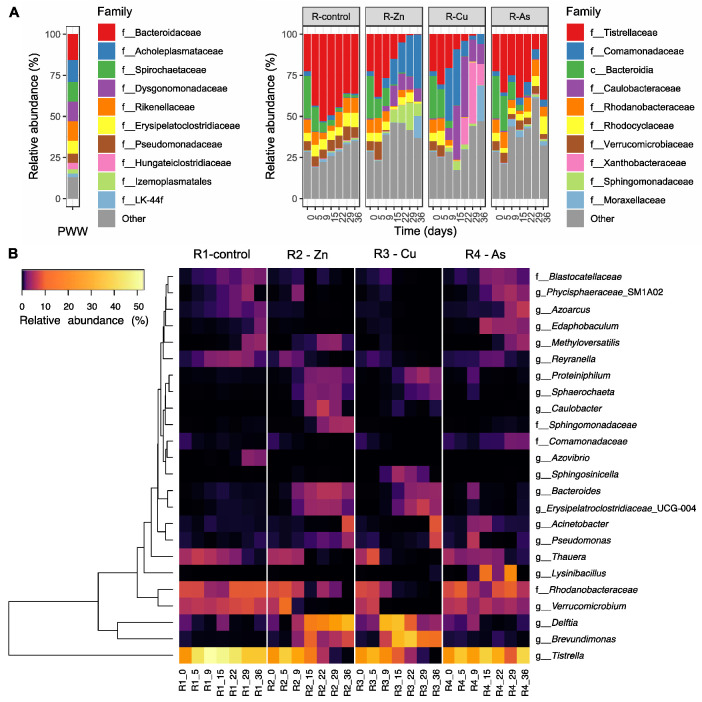
Bacterial community composition of the 10 most abundant taxa in the feed and PBRs at the family taxonomic level (**A**). The composition of the feed before dilution is shown (PWW, left). In the case of the PBRs, changes over time are shown for each reactor (right). Heatmap of bacteria differentially distributed among PBRs (**B**). The rows represent the bacterial genera found in the SIMPER analysis grouped according to the similarity of their relative abundance patterns. The columns represent the samples, which are labeled with the reactor and day. Cell color indicates bacteria relative abundances.

The bacterial composition was very different between the PBRs and feed (Figure 4A and Figure S6). PWW, used as the feed, was dominated by the phyla *Bacteroidota* and *Firmicutes* and to a lesser extent by *Spirochaetota* and *Proteobacteria*. The most abundant classes were *Bacteroidia*, *Bacilli*, *Spirochaetia*, and *Clostridia*. Although *Bacteroidota* was also predominant in the microbiome of the PBRs (on average over 20% of the community), these were dominated by the phylum *Proteobacteria* with more than 60% of relative abundance. *Proteobacteria* was mainly composed of the *Alpha-* and *Gamma-proteobacteria* classes (Figure S6A,B).

Differences in bacterial taxa also appeared between PBRs. Some bacterial communities that increased during exposure to Zn, Cu, and As in phase II with respect to the control reactor were quite abundant in the PWW used as the feed. For instance, at the phylum level, *Firmicutes* increased in relative abundance in all doped PBRs compared to the control reactor. These *Firmicutes* mainly belonged to the *Bacilli* class, and to a lesser extent to the *Clostridia* class in R2-Zn and R3-Cu (Figure S6A,B). In general, the bacterial taxa in the PBRs doped with Zn and Cu markedly changed their abundances with respect to the R1-control and R4-As (Figure 4A). The *Caulobacterales, Bacteroidales* and *Burkholderiales* orders and the *Caulobacteraceae, Bacteroidaceae and Comamonadaceae* families increased more in R2-Zn and R3-Cu than in the rest of the PBRs during phase II (Figure 4A and Figure S6C, 9 to 29 days). By contrast, the *Tistrellales*, *Verrucomicrobiales* and *Xanthomonadales* orders and the *Tistrellaceae*, *Verrucomicrobiaceae* and *Rhodanobacteraceae* families declined through out phase II in R2-Zn and R3-Cu, but recovered in the R1-Control and R4-As.

Although the bacterial composition was more similar between R4-As and the R1-control, there were some differences between them. For example, the *Pseudomonadales* order and the *Comammonadaceae* family increased in R4-As compared to the control (Figure 4A and Figure S6C). In addition, some taxa were dominant in only one reactor, such as the *Sphingomonadaceae* family in R2-Zn and the *Xanthobacteraceae* family in R3-Cu (Figure 4A). During phase III after exposure to HMs (day 36), the bacterial communities in the PBRs exposed to Zn and Cu did not recover the composition profiles of phase I (days 0 to 5).

### 3.5. Effects of the Addition of Metal(loid)s on Bacterial Communities

To analyze the development of different community assemblies in the PBRs under Zn, Cu, and As exposition, a SIMPER analysis was performed among the bacterial communities of the PBRs to identify bacterial genera that were differentially distributed among the PBRs during phases II and III. A total of 48 bacterial species, which belonged to 24 genera, were found to be differentially distributed among the PBRs (Figure 4B, Table S6). The relative abundance of several bacteria decreased during phase II. For example, the genus *Azovibrio* decreased in the presence of all tested toxic elements. However, the distribution of most of the bacteria found in the SIMPER analysis were toxicant-dependent; many bacteria were negatively affected by both Zn and Cu and for some the effect was stronger in the presence of Cu rather than Zn. *Tistrella mobilis* represented 44 ± 7% of the bacterial community in the R1-control during phase II, but it progressively decreased in R2-Zn and R3-Cu during HMs exposure to less than 1% on day 29 in both PBRs (Figure 4B). Similar behavior was observed for the genera *Verrucomicrobium*, *Thauera*, *Methyloversatilis*, and uncultured genera of the *Rhodanobacteraceae* family.

By contrast, several bacteria were favored by the presence of one or more HMs. For example, in R3-Cu, the genus *Delftia* rapidly reached relative abundances of above 30% after the addition of the metal and it was later replaced by the genus *Brevundimonas*. In the presence of Zn, *Delftia* also increased, although its increment was slower than in R3-Cu and it was maintained during phases II and III. Furthermore, *Brevundimonas* was not as predominant as in R3-Cu. *Bacteroidetes*, *Proteiniphilum*, *Sphaerochaeta*, and *Erysipelatoclostridiaceae* UCG-004 genera increased their relative abundances in the presence of both Cu and Zn as well. Some bacteria only incremented under the presence of one metal. For example, *Caulobacter* and an unknown genus of the family *Sphingomonadaceae* were favored by Zn, whereas *Sphingosinicella* initially increased only after the addition of Cu.

Although the overall bacterial composition of R4-As was similar to the R1-control, some genera increased in abundance in the presence of As. For example, *Lysinibacillus*, *Edaphobaculum*, *Azoarcus*, and an uncultured genus of the *Comamonadaceae* family increased in R4-As during phase II. The temporal trend observed for the genera *Acinetobacter* and *Pseudomonas* was toxicant-depended. In R4-As, both taxa increased slightly in the first days of exposure to As and decreased afterwards. By contrast, in R2-Zn and R3-Cu, both genera increased during phase III.

**Figure 5:**
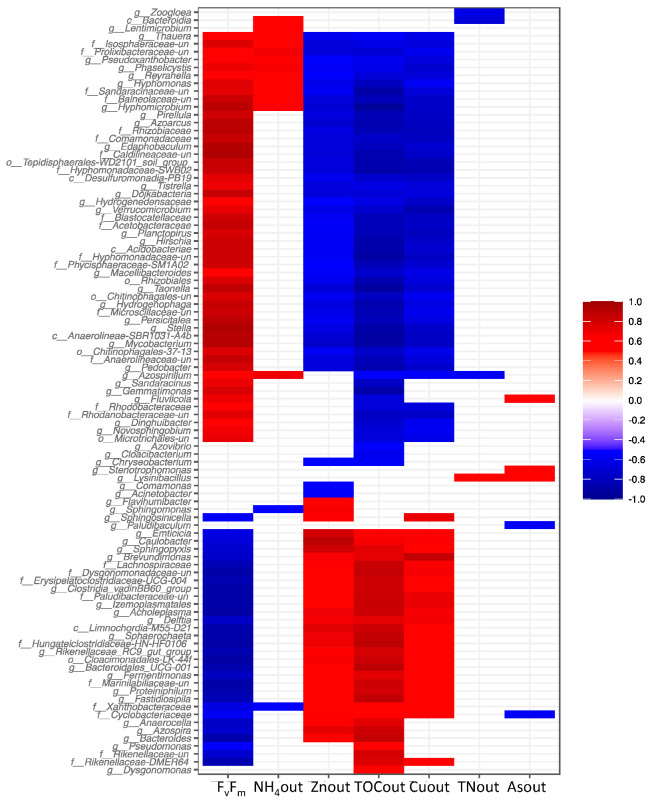
Spearman’s rank correlations are observed between relevant reactor operational variables and bacteria genera. Only taxa with at least a relative abundance >1% are shown. F_v_F_m_: ratio of variable to maximum fluorescence, (NH_4_-Zn-TOC-Cu-TN and As) out: ammonium, zinc, total organic carbon, copper, total nitrogen and arsenic concentration in the effluent. Only correlations that are significant (adjusted *p*-values < 0.05) are shown. See Supplementary Table S7 for full results. g__uncultured corresponds to uncultured or unknown genera.

Finally, Spearman rank correlations were calculated between bacteria genera and the relevant operational variables (Figure 5, for full results see Table S7). Two main trends were observed. First, taxa positively correlated with F_v_/F_m_ and negatively correlated with high levels of TOC, Zn, and Cu in the effluent, for instance, the *Verrucomicrobium* (ρ: F_v_/F_m_: 0.778, TOC: −0.738, Cu: −0.860, Zn: −0.611), *Tistrella* (ρ: F_v_/F_m_: 0.772, TOC: −0.648, Cu: −0.698, Zn: −697), *Blastocatellaceae* (ρ: F_v_/F_m_: 0.865, TOC: −0793, Cu: −0.745, Zn: −0.574), *Rhodanobacteraceae* uncultured (ρ: F_v_/F_m_: 0.775, TOC: −0751, Cu: −0.759), and *Reyranella* (ρ: F_v_/F_m_: 0.691, TOC: −0703, Cu: −0.702) genera. On the other hand, another group of bacterial taxa were positively correlated with HMs and high TOC concentrations in the effluent and negatively correlated to F_v_/F_m_ ratio, for instance the *Delftia* (ρ: F_v_/F_m_: −0.741, TOC: 0.741), *Brevundimonas* (ρ: F_v_/F_m_: −0.727, TOC: 0.727), and *Proteiniphilum* (ρ: F_v_/F_m_: −0.838, TOC: 0.814) genera. Most of the bacteria that were differentially distributed in the Zn and Cu doped PBRs (Figure 4B), belonging to any of those two groups. Interestingly, among bacterial taxa characteristic of R4-As found in the SIMPER analysis, only *Lysinibacillus* was correlated with As (ρ: 0.644). This genus was also correlated with high TN in the effluent (ρ: 0.643). The other bacteria characteristic of R4-As (i.e., *Edaphobaculum, Azoarcus*, and *Comamonadaceae*) were correlated with high values of F_v_/F_m_ and negatively correlated with TOC, Zn, and Cu in the effluent (Table S7). These genera were among the bacteria most strongly correlated with F_v_/F_m_ values.

## 4. Discussion

This study has shown that the current maximum concentrations of Zn, Cu, and As compromise TOC and TN removal efficiencies. Moreover, the PBRs microbial communities of microalgae and bacteria are largely affected by these pollutants, which are responsible for 81.4% of the bacterial community variance. The higher similarity of R4-As performance and microbial community to the R1-control compared to R2-Zn and R3-Cu could be related to the differences in the doped concentrations of each metal(loid) because the As concentration applied was three orders of magnitude smaller (500 μg/L) than the Zn and Cu concentrations (100 mg/L). These different concentrations were chosen within the highest levels reported in PWW to date to analyze the most extreme but realistic scenarios on reactor performance and microbiomes.

### 4.1. Negative Effect of Zn and Cu on PBRs Performance Associated with Changes in Microbial Communities

The addition of Zn and Cu caused a clear inhibition of growth and photosynthesis capacity of *Chlorella* sp. which never recovered after the addition of HMs. Previous studies have reported that Zn and Cu decrease the microalgal growth by inhibiting chlorophyll synthesis and inactivating photosystem II reaction centers, resulting in an inhibition of photosynthetic activity [13]. Although Cu caused a slower decrease in F_v_/F_m_ ratio than Zn, in this study, the final results obtained in phase II for the F_v_/F_m_ ratio in PBRs R2 and R3 indicate that both Zn and Cu caused damage to the photosynthetic apparatus compared to the R1-control, which remained within the expected values for these cultivation systems (>0.4, [41]). It was previously reported that growth and chlorophyll content in the genus *Chlorella* decreased at concentrations between 2–3 mg/L Cu and 20–25 mg/L Zn in both axenic culture and swine wastewater in high-rate algae ponds [18,42]. Therefore, our results were not surprising; in fact, it provides new evidence on the inhibition of *Chlorella* sp. at 100 mg/L of Zn and Cu in microalgae–bacteria PBRs for PWW.

In addition, the high inhibition of microalgae in R2-Zn and R3-Cu could be due to a large absorption of Zn and Cu in the algae–bacteria biomass, causing cell and photosynthetic damage. The results obtained for biomass uptake and removals of Zn (>140 mg/g; >75%) and Cu (>120 mg/g; >80%) were high compared to previously reported values. In general, the uptakes by different species of the genus *Chlorella* using non-living cells range from 6.42 to 43.41 mg/g for Zn and from 1.8 to 108 mg/g for Cu [12]. Moreover, it has been reported that the uptake of different bacterial species can range from 92 to 172 mg/g at 100–150 mg/L of Zn and between 32–33 mg/g at 100 mg/L of Cu [15,43]. The removal of Zn and Cu by a monoculture *Chlorella minutissima* was 62% at 100 mg/L of Zn and 30% at a concentration near 100 mg/L of Cu. A consortium co-culture of *Chlorella vulgaris* and bacteria provided similar Cu removal efficiencies than those obtained in this work (78.7% from synthetic wastewater with 100 mg/L of Cu) [44].

In parallel to *Chlorella* sp. inhibition, Zn and Cu decreased the TOC removal efficiencies in PBRs R2 and R3. The death of microalgae and other organisms due to HMs exposure might cause the release of the cell content into the PBRs, incrementing the TOC concentrations in the effluent. However, the partial increase in TOC removal efficiencies during phase III, while *Chlorella* was not able recover growth, suggests that changes in TOC removal efficiencies could also be attributed to low bacterial cell death or changes on heterotrophic bacteria, which might have partially recovered their TOC removal capacities during phase III after suffering a negative impact during phase II.

HMs largely affected bacterial communities without recovery after exposure. The microorganisms responsible for the community changes under Zn and Cu expositions were distributed in two main groups. First, microorganisms negatively affected by HMs, which were related to an adequate performance of the PBRs and the good state of the microalgae and that were characteristic of the R1-control. Second, the bacteria favored by the HMs and related to increments of nutrient concentrations in the doped PBRs effluents. In the first case, the heterotrophic bacteria, although able to thrive under standard operational conditions found in the R1-control, are likely inhibited by the high concentrations of HMs that might exert a toxic effect and thus cause a decrease in these taxa and nutrient removals, such as TOC. Among them, the genus *Tistrella* stood out for its abundance in the R1-control (more than 40% during phase II) but also for its decrease in the presence of Zn and Cu without recovery during phase III. Information on this genus is scarce, although some authors have indicated the potential of some strains, isolated from soils and wastewater, to produce and degrade polycyclic aromatic hydrocarbons [45,46]. More recently, SEM analysis showed *Tistrella* sp. cells associated on the cell surface of the microalga *Chlorella vulgaris* and within aggregates of extracellular structures [47]. Based on this, the low growth of *Chlorella* in the PBRs exposed to Zn and Cu could be related to the decrease in *Tistrella* due to the low production of exopolysaccharides by this microalga, which would reduce the availability of nutrients for this bacterium. Other heterotrophic bacteria commonly found in wastewater treatment microbiomes that were also negatively affected by HMs and negatively correlated with TOC were the *Verrucomicrobium* genus, which appear to be an important group within the normal operation of PBRs. Recently, a decrease in the relative abundance of this genus has been related to a higher concentration of TOC (lower removal) in raceway PBRs at pilot scale treating urban wastewater [41]. Another bacterial population that was affected by HMs was the *Blastocatellaceae* family, which has been associated with hydrocarbon degradation at a petrochemical wastewater treatment plant [48]. Other bacteria characteristic of wastewater treatment that was affected by Zn or Cu could contribute to lower TOC removal in each PBRs. For example, *Thauera* has been identified in several types of wastewater treatments, including in microalgae–bacteria photobioreactors treating swine wastewater [10]. It is capable of heterotrophic denitrification using a variety of organic compounds, including extracellular polymeric substances, as carbon sources while respiring nitrate or nitrite [49], it has also been associated with the heterotrophic nitrification–aerobic denitrification process [50].

By contrast, the group of bacteria that thrived under the exposition to Zn and Cu are characterized by versatile metabolisms with potential capacities to resist HMs. These microorganisms likely replaced the heterotrophs that decayed during phase II. *Delftia* and *Brevundimonas* (of the families *Comamonadaceae* and *Caulobacteraceae*, respectively) increased markedly in the presence of both HMs. It is already known that some *Delftia* strains are highly resistant to HMs as well as metal biosorption capabilities. For example, *D. lacustris* LZ-C, isolated from a petrochemical wastewater discharge site, are resistant to Zn, chromium, mercury, lead and cadmium and degrade various aromatic compounds [51]. *Delftia* sp. B9, are potentially suitable for the bioremediation of cadmium-contaminated paddy soils [52] and the strain-MS3 can be used in water and wastewater as an efficient biosorbent of metal ions [53]. Moreover, several strains of *Brevundimonas* are known for their capacity of biosorption of HMs [54]. *Proteiniphilum* is another abundant genus that was favored by Zn and Cu. *Proteiniphilum* has been associated with antimicrobial and metal resistance genes in composting piles [55]. Other bacterial genera that increase to a lesser extent in R2-Zn and R3-Cu are *Sphaerochaeta*, which have been described in landfill leachate environments with cellulolytic activity and correlated with the presence of heavy metals [56]; *Bacteroides*, which are dominant in wastewater with several pathogenic strains resistant to antibiotics and Zn [57]; and *Erysipelatroclostridiaceae*, which has been found in pig excrement [58]. Although these bacterial groups have a diverse metabolism with the capacity to degrade complex organic compounds, in this study, a lower removal of organic compounds was observed; therefore, the presence of these taxa could contribute to the higher removal of HMs by the biomass obtained in this study.

The bacteria favored by the addition of HMs are part of large taxonomic groups typically found in piggery wastewaters. For example, the families *Comamonadaceae*, *Rhodocyclaceae*, *Rikenellaceae*, *Saccharospirillaceae, Caulobacteraceae* and *Sphingomonadaceae* are found in animal excreta, sewage water and PWW [10,41,59,60]. This observation highlights that the source of the bacteria that are filling the niches that become available as the PBRs inhabitants are affected by the pollutants is the PWW rather than dormant or rare bacteria already present in the PBRs. The influence of immigration in activated sludge systems has been already demonstrated [61], which may allow for improving and favoring some operational conditions of the reactor to increase the presence of essential taxa in the elimination of nutrients and pollutants for better reactor performance.

Reactor performance was different between R2-Zn and R3-Cu. While only TOC removal efficiencies were altered in R2-Zn, nitrogen removals were lower, while TSS and oxygen concentrations were higher in the Cu-doped reactor than in the R1-control. The significant increase in TSS may be due to a complexation of Cu and organic matter, which caused flocculation and thus a higher total solids content in the effluent [62]. The higher oxygen concentrations in R3 than in R2 could be attributed to a combined effect between a slower inhibition by Cu of the photosynthesis process (slower decrease in the F_v_/F_m_ parameter with Cu than with Zn), and a higher toxic effect of Cu than of Zn on heterotrophic bacteria. Previous studies have reported that oxygen concentration in PBRs reflects the interaction between microalgae and bacteria responsible for organic matter degradation [18]. In addition, Cu also affected TN removal. In the microalgae–bacteria reactor systems, TN removal occurs mainly by assimilation into the biomass and/or nitrification/denitrification processes [63]. Some species with N-removal capabilities decreased under Cu but not Zn exposition, which might explain the difference in TN removal between the two PBRs. For example, the *Rhodanobacteraceae* family and the *Methyloversatilis* genus have been positively correlated with TN removal as they are capable of denitrification [50,64]. Moreover, the genus *Reyranella* can reduce nitrate to nitrite in soil [65]. Therefore, their removal from R3-Cu could increase inorganic nitrogen and contribute to the drop of TN removals. Some other bacteria were favored in R2-Zn but not in R3-Cu PBRs, such as the family *Sphingomonadaceae* and the genus *Caulobacter*. The family *Sphingomonadaceae* has been correlated with lower nitrate concentrations [41]. On the other hand, the genus *Caulobacter* have genes associated with nitrate reduction [66]. Previous studies indicate that Cu and Zn concentrations have different effects on nitrifying/denitrifying bacteria; however, Cu has been found to have a greater inhibitory effect than Zn, with denitrification being more susceptible than nitrification under aerobic conditions [67,68]. Therefore, this work extends our understanding of the effects of HMs on bacteria involved in nitrogen removal in microalgae–bacteria PBRs. In particular, denitrification, favored during the dark cycle of the PBRs, is one of the main nitrogen removal mechanisms in wastewater treatment and its decay in the presence of Cu would contribute to low nitrate reduction, leading to poor water quality.

### 4.2. The impact of As on the Removal of Nutrients and the Microbiome of Reactor

In contrast to Zn and Cu, the addition of As favored the growth of *Chlorella* sp. which had a physiological state close to those recorded in healthy microalgae cultures (F_v_/F_m_ ratio > 0.6, [26]). These observations indicate that the concentration of As used in this research (500 μg/L) did not affect growth or induce damage to the photosynthetic activity of microalgae. Indeed, previous studies mentioned that several species of *Chlorella* have a high tolerance to toxic concentrations of As, such as 500 mg/L [14] and 1000 mg/L [21], as a result of detoxification mechanisms [69]. In addition, the higher growth of *Chlorella* sp. in R4-As than in the R1-control could be associated with hormesis in response to toxicants at low concentrations (0.3 to 3.7 mg/L) [69]. Furthermore, the lack of As negative impact on microalgae growth could be related to the low uptake of this metalloid by the biomass, resulting in less cell damage [14,21,70]. In this context, the As uptake (>0.180 mg/g) and removal (~19%) obtained in this study were lower than to those reported for an axenic culture of *Chlorella* sp. which reached an uptake of 0.19 mg/g and a removal of 92% at a lower influent As concentration than in this study (80 μg/L of As) [71]. In addition, a large removal (48.38%) of As was also reported for mixed cultures of *Chlorella vulgaris* and activated sludge bacteria during 10 days of culture at 690 μg/L of As during PWW treatment [6]. These differences may be due to various cultivation conditions, such as the concentration of As and strain used, non-phosphorus limiting conditions, and the high organic matter load present in the PWW, which may affect As removal and reduce adsorption by microalgae and bacteria [70,72].

As with microalgae growth, TOC and NH_4_^+^-N removals were stable in the R4-As reactor. The only detrimental effect of As doping on the performance of PBRs was a reduction in TN removal. Similar to what happened in the Cu-doped reactor, the low TN removal in R4-As could be explained by the elimination of some arsenic-sensitive bacteria with denitrifying activity. However, the taxa affected by As and negatively correlated with nitrogen were different from those affected by Cu. For example, *Pusillimonas* was the only genus that correlated simultaneously and negatively with As and nitrogen (*p*-value: −0.733 for TN and −0.646 for As, Table S7). This genus acts as a denitrifier in polluted water [73]. In addition, co-cultures of *Chlorella sorokiniana* and *Pusillimonas* treating stripped food waste permeate reached high TN removal efficiencies [74]. Other denitrifying genera may be also involved in the low TN removal. For example, the genus *Azoarcus* decreased during phase II in the R4-As reactor. Most of the documented species belonging to this genera were identified as important denitrifiers in wastewater treatment plants [64]. Furthermore, the increase in the relative abundance of *Azoarcus* at the end of phase III could explain the increase in TN removal after As exposure. This observation is consistent with another study, which indicates a considerable capacity of *Azoarcus* to adapt to the toxic effect of As through detoxification mechanisms [75]. Previously, it has been reported that nitrification at 100 mg/L As was not affected in a fluidized-bed reactor with simulated mine water [76]. By contrast, the inhibition of denitrification by As has been reported in shallow lakes and in studies under anaerobic conditions [77]. Therefore, it is not surprising that in this work we have observed alterations in the nitrification/denitrification process in the presence of As, with an impact on nitrogen removal. In fact, this evidence extends the knowledge to microalgae–bacteria PBRs treating PWW.

Bacterial populations that increased during As exposure were likely playing a fundamental role in reactor performance during the exposition to the metalloid. In this context, the genera *Lysinibacillus* and *Acinetobacter* and the family *Comamonadaceae* increased their abundance relative to R4 with respect to the other reactor. *Lysinibacillus* is a key contributor in the degradation and formation of dissolved organic matter during composting [78] and some strains have As resistance and detoxification capacities [79]. Likewise, *Acinetobacter* and *Comamonadaceae* have been found in microalgal co-cultures and in PBRs treating wastewater with high biomass productivity, and their role has been attributed to the beneficial exchange of carbon dioxide and oxygen between algae and bacteria, as well as to the synthesis of vitamins for microalgal growth [41,80,81]. In addition, other taxa that increased in the presence of As, such as the *Gemmatimonadaceae* and *Saccharimonadales* families, can degrade organic matter, remove phosphorus, and act as indicators of contaminated water, as well as carry out nitrification processes in the soil and infiltration systems for underground wastewater [59,60]. Therefore, the presence of these taxa could explain the good reactor performance and growth of the microalgae in the presence of As.

## 5. Conclusions

Microalgae–bacteria photobioreactors are a viable and sustainable alternative for the treatment of wastewaters produced in pig farms. However, high concentrations of Zn, Cu, and As in piggery wastewater significantly affect the performance of treatment photobioreactors, the quality of the effluents, and biomass generated. Zn and Cu are accumulated in the microalgae–bacteria biomass, significantly affecting the growth of microalgae and, reducing the amount of heterotrophic bacterial communities capable of oxidizing organic compounds, resulting in low carbon removal from the wastewater. In addition, Cu and As had a significant impact on denitrifying bacteria populations, which may have contributed to reduce the nitrogen removal. Despite its higher toxicity, As had a lower impact on the reactor microbiome than Zn and Cu. This low impact could be related to differences in concentrations present in the wastewater at the inlet, but also to the development of arsenic resistance mechanisms by microalgae and bacteria, which resulted in an increase in microalgae growth, a low uptake of this metalloid in the biomass, and in a high percentage of As leaving in the treated water. The presence of metal(loid)s in piggery wastewater compromises the overall performance and microbiome of the photobioreactors in an irreversible way, contributing to the environmental and health impact of the discharge of effluent. Therefore, these metal(loid)s should be monitored for some applications, such as the use of biomass as animal feed or fertilizer and the treated water for irrigation.

## Figures and Tables

**Table 1:** Physicochemical composition of 5% *v/v* diluted PWW used as feed for the sets of experiments A and B. Values correspond to the mean ± standard deviation obtained of the feed samples analyzed every two days during the whole experiment.

Parameters (Unit)	Experiment A	Experiment B
Total suspended solid (TSS in g/L)	0.42 ± 0.1	0.3 ± 0.02
Total organic carbon (TOC in mg/L)	713 ± 26	652 ± 27
Total nitrogen (TN in mg/L)	256 ± 11	247 ± 11
Ammonia nitrogen (NH_4_^+^-N in mg/L)	141 ± 6	164 ± 10
Total phosphorus (TP in mg/L)	3.8 ± 0.21	3.6 ± 0.17
Zinc (Zn in mg/L)	0.98 ± 0.63	0.78 ± 0.23
Copper (Cu in mg/L)	0.28 ± 0.15	0.25 ± 0.05
Arsenic (As in μg/L)	2.04 ± 0.19	1.94 ± 0.22

## Data Availability

Not applicable.

## Supplementary Materials

The following supporting information can be downloaded at: https://www.mdpi.com/article/10.3390/biology11081176/s1, Figure S1: Concentrations of nutrients and metal(loid)s in the effluent for the set of experiment A. TOC (A), TN (B), NH_4_^+^-N (C), Zn (D), Cu (E), and As (F). Vertical dashed lines indicate the change of phase; Figure S2: Microalgal growth parameters during experimentation time for experiments A. Cell count (A), maximum quantum yield of PSII by F_v_/F_m_ ratio (B), and TSS (C) in the R1, R2, R3 and R4 PBRs, corresponding to control and PBRs fed with Zn, Cu, and As, respectively; Figure S3: Dissolved oxygen concentration in the photobioreactors (R1, R2, R3, and R4) for experiment B; Figure S4: Alpha bacterial diversity indices. Richness and diversity indices increase as the number of species and their diversity increase. Evenness ranges from 0 (no community uniformity) to 1 (complete community uniformity). Left column: temporal changes through all experimental phases of richness (A), diversity (C), and evenness (E). Changes of experimental phases are indicated by vertical lines. Right column: boxplots of the data distribution of diversity indices for each reactor during phase II. Boxes indicate the first and third quartile, which is the interquartile range (IQR). The thick inner line indicates the median. Whiskers mark the most distant data within the upper and lower 1.5 IQR distance. Dots indicate other data beyond those limits. Pair-wise Wilcoxon tests were used to compare the indices between reactors during the exposure to metals. Tests that were statistically significant (*p*-value < 0.05) are indicated with an asterisk, ns: not significant; Figure S5: Variation in community composition among PBRs over time, as well as the raw PWW are observed. Non-Metric Multidimensional Scaling Analysis (NMDS) based on the Bray–Curtis distance measure. Prior to the analysis, bacterial species that are not present in more than 0.1% relative abundance in any sample were removed; Figure S6: Changes in the bacterial composition over time of the feed and the microbiomes of each reactor at the taxonomic levels of phylum (A), class (B), and order (C). The composition of the feed before dilution is shown (left, PWW). In the case of PBR, changes over time are shown for each reactor (right). The 10 most abundant are shown; Table S1: The effect of the Zn, Cu, and As on the performance of the PBRs for experiment A and B. Analysis of variance (ANOVA) and Tukey’s honestly significant difference (HSD) multiple range tests (with confidence level of 95%) were carried out to identify significant differences among PBRs during steady-state of phase II (from day 15 to 29). Statistically significant are indicated in bold; Table S2: Averaged diversity indices. The means and standard deviations of the reactors were calculated for the full experiment. For the raw PWW sample, one observation was obtained; Table S3: PERMANOVA global test for Reactor factor; Table S4: PERMANOVA pair-wise comparisons. Comparisons that are statistically significant with a 5% level of confidence are indicated with an asterisk; Table S5: tbRDA data. Variance explained by the constrained ordination. Variables included in the model are Cu. Zn, and As concentrations in the feed (3 variables) and in both the liquid and solid phases of the effluent (6 variables); Table S6: SIMPER results; Table S7: Spearman’s correlations between selected operational variables and bacterial genera. Only significant correlations (adjusted *p*-values < 0.05) are indicated. Stronger correlations |0.70| are marked with colored cells: blue for negative and red for positive values.

## Abbreviations

The following abbreviations are used in this manuscript:

PWW	Piggery wastewater
HMs	Heavy metals
PBRs	Photobioreactors
TSS	Total suspended solid
TOC	Total organic carbon
TN	Total nitrogen
NH_4_^+^-N	Ammonia nitrogen
TP	Total phosphorus
HRT	Hydraulic retention time
T	Temperature
DO	Dissolved oxygen
PSII	Photosystem II
F_v_/F_m_	Ratio of variable to maximum fluorescence
tbRDA	Transformation-based redundancy analysis
NMDS	Non-metric multidimensional scaling
SIMPER	Similarity percentages
