# Supplementary material for: Current Concentrations of Zn, Cu, and As in Piggery Wastewater Compromise Nutrient Removals in Microalgae–Bacteria Photobioreactors Due to Altered Microbial Communities
Source: Biology (Basel). 2022 Aug 5;11(8):1176. doi: 10.3390/biology11081176 (PMC9405037; doi:10.3390/biology11081176)
Supplement: Supplementary file 1 [file biology-11-01176-s001.zip › biology-1833727-supplementary.pdf]

Current concentrations of Zn, Cu, and As in piggery wastewater compromise nutrient removals in microalgae−bacteria photobioreactors due to altered microbial communities

Javiera Collao[1,2], Pedro Antonio García-Encina[1,2], Saúl Blanco[3], Silvia Bolado-Rodríguez[1,2,*] and Nuria Fernandez-Gonzalez[1,2, 4]

[1] Department of Chemical Engineering and Environmental Technology. Univer-sity of Valladolid, Dr. Mergelina s/n, 47011, Valladolid, Spain; javiera.collao@alumnos.uva.es (J.C.); pedoantonio.garcia@uva.es (P.G.-E.); nuria.fer.gonzalez@gmail.com (N.F.-G.)
[2] Institute of Sustainable Processes (ISP). University of Valladolid, Dr. Merge-lina s/n, 47011, Valladolid, Spain
[3] Department of Biodiversity and Environmental Management. University of León, 24071, León, Spain.
[4] Department of Systems Biology, Spanish Center for Biotechnology, CSIC, C/ Darwin nº3, 28049 Madrid, Spain

– SUPPLEMENTARY DATA –

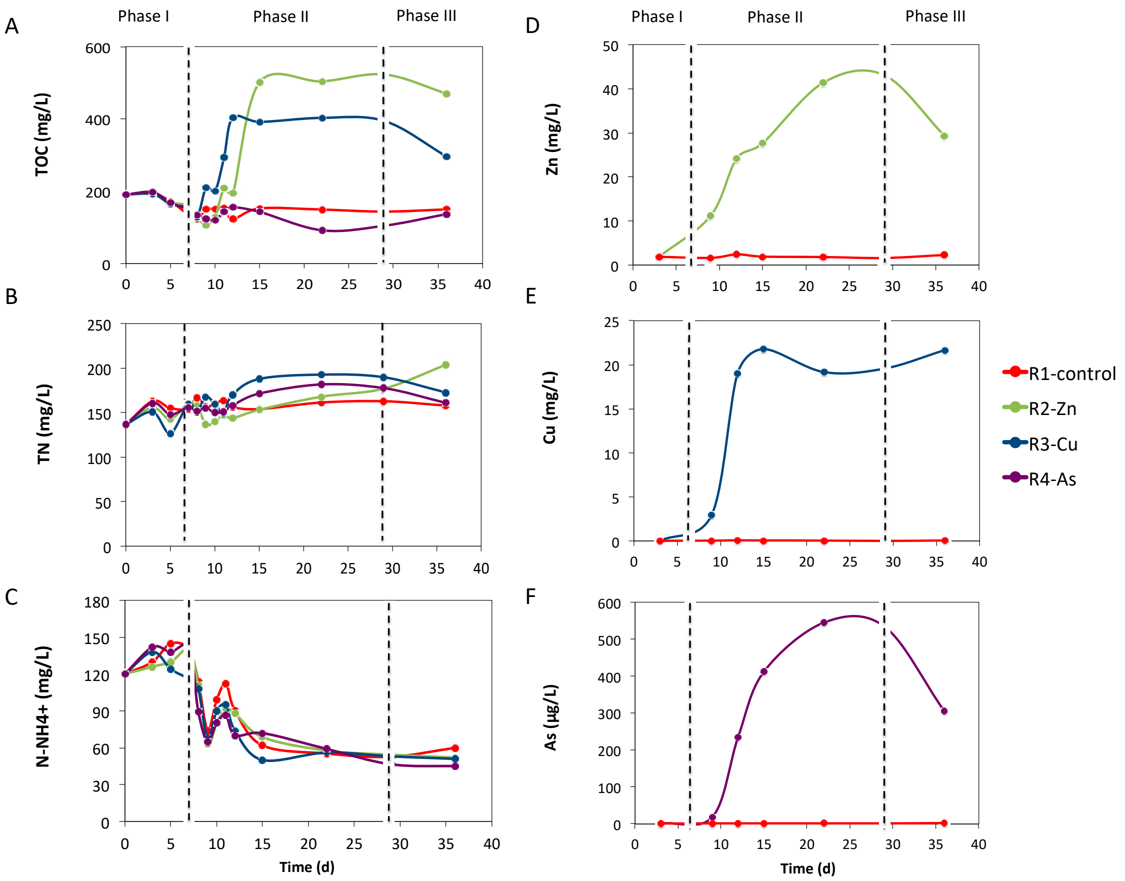

Figure S1. Concentrations of nutrients and metal(loid)s in the effluent fot the set of experiment A. TOC (A), TN (B), NH_4^+-N (C), Zn (D), Cu (E), and As (F). Vertical dashed lines indicate the change of phase.

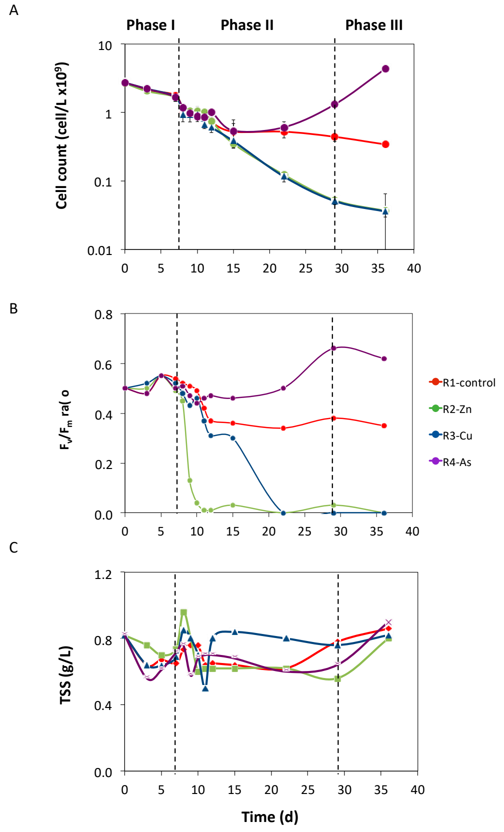

Figure S2. Microalgal growth parameters during experimentation time for experiments A. Cell count (A), maximum quantum yield of PSII by F_v/F_m ratio (B), and TSS (C) in the R1, R2, R3 and R4 PBRs, corresponding to control and PBRs fed with Zn, Cu, and As, respectively.

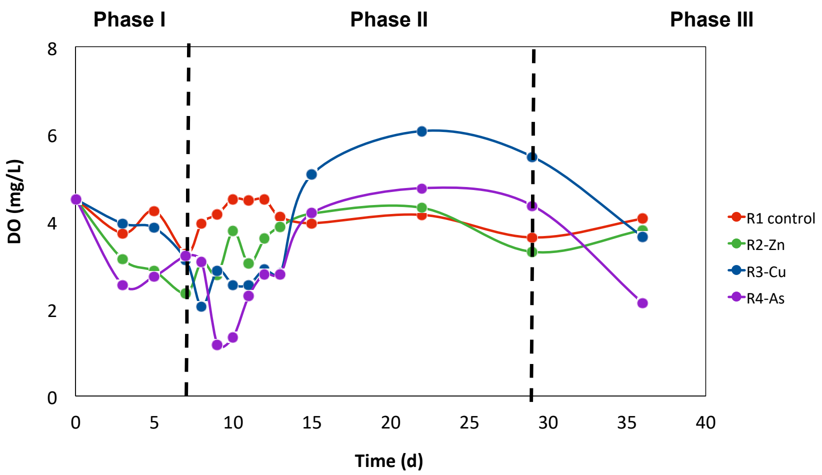

Figure S3. Dissolved oxygen concentration in the photobioreactors (R1, R2, R3, and R4) for experiment B.

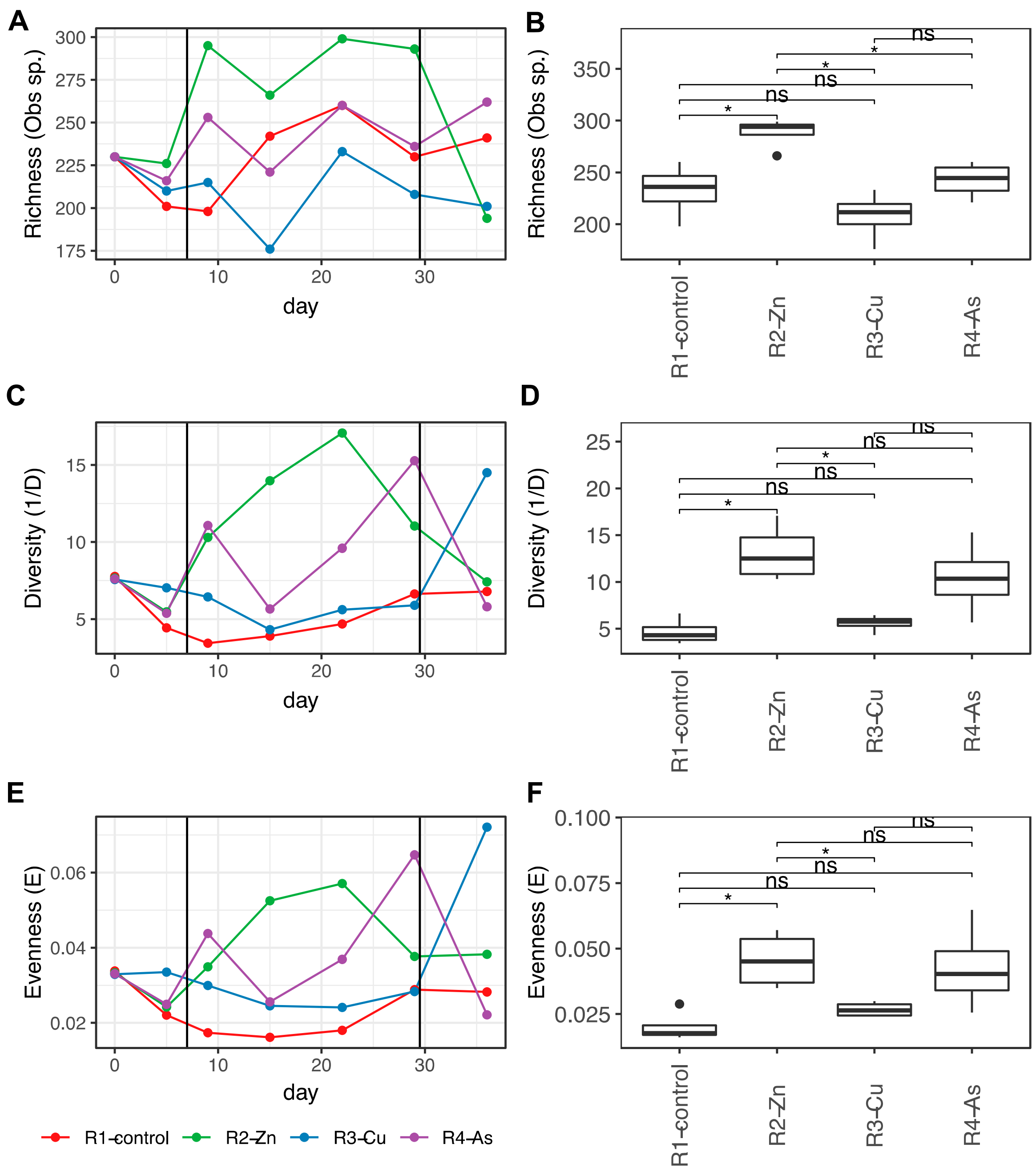

Figure S4. Alpha bacterial diversity indices. Richness and diversity indices increase as the number of species and their diversity increase. Evenness ranges from 0 (no community uniformity) to 1 (complete community uniformity). Left column: temporal changes through all experimental phases of: richness (A), diversity (C), and evenness (E). Changes of experimental phases are indicated by vertical lines. Right column: boxplots of the data distribution of diversity indices for each reactor during phase II. Boxes indicate the first and third quartile, which is the interquartile range (IQR). The thick inner line indicates the median. Whiskers mark the most

distant data within the upper and lower 1.5 IQR distance. Dots indicate other data beyond those limits. Pair-wise Wilcoxon tests were used to compare the indices between reactors during the exposure to metals. Tests that were statistically significant (p-value < 0.05) are indicated with an asterisk, ns: not significant.

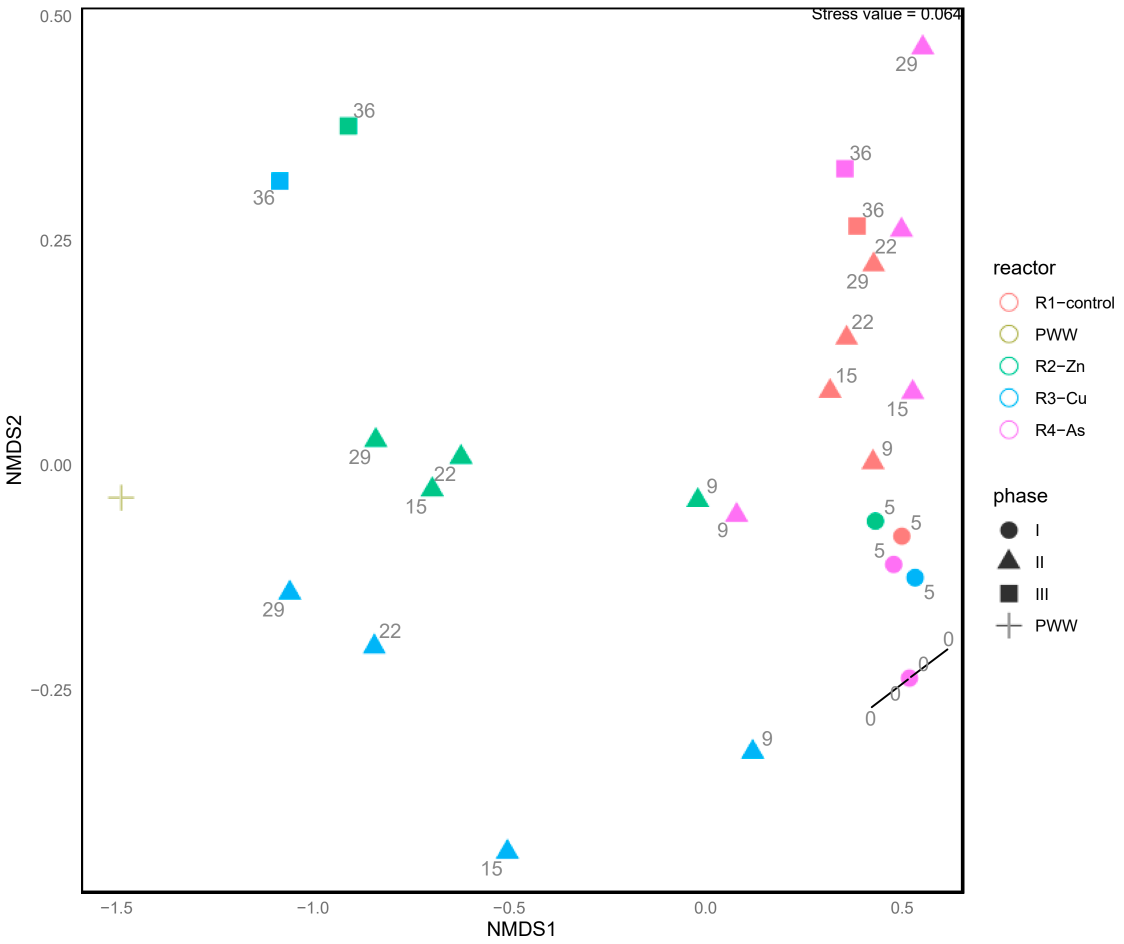

Figure S5. Variation in community composition among PBRs over time, as well as the raw PWW are observed. Non-Metric Multidimensional Scaling Analysis (NMDS) based on the Bray−Curtis distance measure. Prior to the analysis, bacterial species that are not present in more than 0.1% relative abundance in any sample were removed.

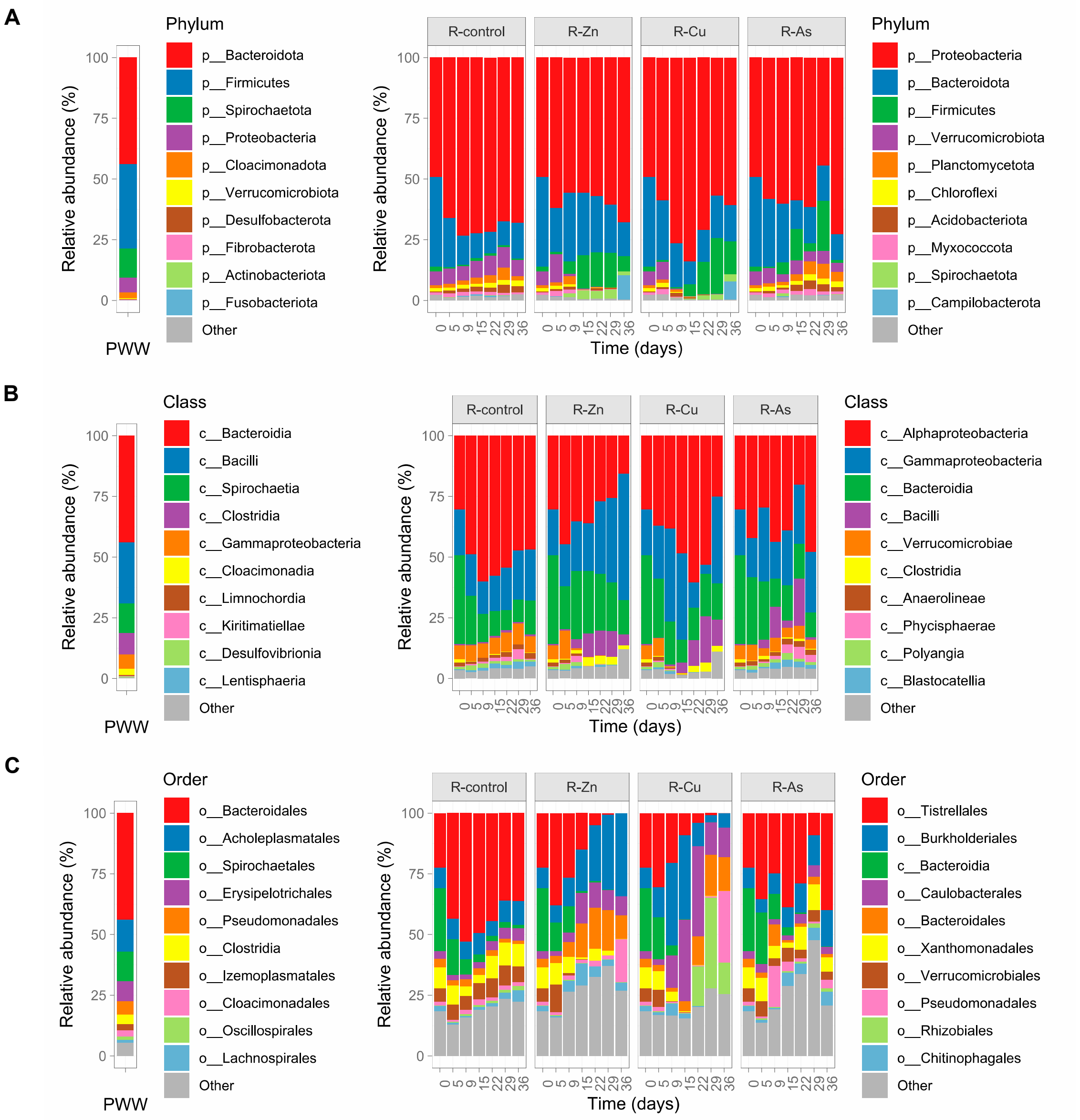

Figure S6. Changes in the bacterial composition over time of the feed and the microbiomes of each reactor at the taxonomic levels of phylum (A), class (B), and order (C). The composition of the feed before dilution is shown (left, PWW). In the case of PBR, changes over time are shown for each reactor (right). The 10 most abundant are shown.

Table S1. The effect of the Zn, Cu, and As on the performance of the PBRs for experiment A and B. Analysis of variance (ANOVA) and Tukey's honestly significant difference (HSD) multiple range tests (with confidence level of 95%) were carried out to identify significant differences among PBRs during steady-state of phase II (from day 15 to 29). Statistically significant are indicated in bold.

**Experiment A**								
		TOC		TN		NH_4^+-N		SST
		Eff (mg/L)	RE (%)	Eff (mg/L)	RE (%)	Eff (mg/L)	RE (%)	(g/L)
ANOVA	F-ratio	399.1	354.16	10.69	8.33	0.52	0.36	7.47
	p-value	0.0000	0.0000	0.0036	0.0076	0.681	0.786	0.011
± Limits		30.21	0.0517	13.53	0.046	20.58	0.128	0.143
Tukey HSD (difeference)	R1-control - R2-Zn	**-360.66**	**0.4**	-6.66	0.01	-3.66	0.016	0.08
	R1-control - R3-Cu	**-247.66**	**0.27**	**-31**	**0.09**	3.66	-0.02	-0.12
	R1-control - R4-As	12.33	-0.04	**-17.66**	**0.046**	-2.66	0.006	0.04
	R2-Zn - R3-Cu	**113**	**-0.126**	**-24.33**	**0.08**	7.33	-0.04	**-0.2**
	R2-Zn - R4-As	**373**	**-0.446**	-11	0.036	1	-0.01	-0.04
	R3-Cu - R4-As	**260**	**-0.32**	13.33	-0.043	-6.33	0.03	**0.16**

**Experiment B**									
		TOC		TN		NH_4^+-N		SST	DO
		Eff (mg/L)	RE (%)	Eff (mg/L)	RE (%)	Eff (mg/L)	RE (%)	(g/L)	(mg/L)
ANOVA	F-ratio	30.79	30.17	8.15	16.77	2.22	2.19	8.46	9.85
	p-value	0.0001	0.0001	0.008	0.000	0.163	0.166	0.007	0.005
± Limits		39.97	0.0500	21.47	0.070	14.9	0.081	0.152	1.1
Tukey HSD (difeference)	R1-control - R2-Zn	**-96.66**	**0.12**	-1.66	0.003	7.33	-0.033	0.11	-0.02
	R1-control - R3-Cu	**-73**	**0.11**	**-25.66**	**0.116**	7	-0.013	-0.13	**-1.63**
	R1-control - R4-As	-3.33	0.01	**-22.66**	**0.106**	-2.33	0.03	0.02	-0.52
	R2-Zn - R3-Cu	23.66	-0.02	**-24**	**0.113**	-0.33	0.02	**-0.24**	**-1.61**
	R2-Zn - R4-As	**93.33**	**-0.11**	-21	**0.103**	-9.66	0.063	-0.09	-0.51
	R3-Cu - R4-As	**69.66**	**-0.09**	3	-0.01	-9.33	0.043	0.15	**1.1**

Table S2. Averaged diversity indices. The means and standard deviations of the reactors were calculated for the full experiment. For the raw PWW sample, one observation was obtained.

**Reactors**	**Richness (observed species)**	**Diversity (1/D)**	**Evenness (E)**	**Chao1**	**Diversity_shannon**
R1-Control	229 ± 22	5.4 ± 1.6	0.023 ± 0.007	231 ± 21	2.8 ± 0.3
R2-Zn	257 ± 40	10.4 ± 4.1	0.040 ± 0.012	259 ± 39	3.2 ± 0.4
R3-Cu	210 ± 20	7.3 ± 3.3	0.035 ± 0.017	212 ± 19	3
R4-As	239 ± 17	8.6 ± 3.7	0.036 ± 0.015	243 ± 19	3.1 ± 0.4
Raw-PWW	252	23.1	0.091	252	3.7

Table S3. PERMANOVA global test for Reactor factor.

	Degrees of freedom	Sums of Squares	Mean sums of squares	F.model	R2	Pr(>F)
**Reactor**	3	2.0769	0.69229	6.3476	0.61344	0.001
**Residuals**	12	1.3088	0.10906		0.38656	
**Total**	15	3.3856			1.00000	

Table S4. PERMANOVA pair-wise comparisons. Comparisons that are statistically significant with a 5% level of confidence are indicated with an asterisk.

Pairs	F.Model	R^2	p-value	adjusted p-value	
**R1-control vs. R2-Zn**	13.446215	0.6914567	0.03	0.048	*
**R1-control vs. R3-Cu**	10.236611	0.6304647	0.032	0.048	*
**R1-control vs. R4-As**	2.635032	0.305156	0.062	0.0744	
**R2-Zn vs. R3-Cu**	1.709179	0.221707	0.236	0.236	
**R2-Zn vs. R4-As**	6.963468	0.5371609	0.021	0.048	*
**R3-Cu vs. R4-As**	6.208181	0.5085263	0.023	0.048	*

Table S5. tbRDA data. Variance explained by the constrained ordination. Variables included in the model are: Cu. Zn, and As concentrations in the feed (3 variables) and in both the liquid and solid phases of the effluent (6 variables).

	Inertia[a)]	Proportion	Rank
**Total**	0.38471	1.00000	
**Constrained**	0.31310	0.81386	9
**Unconstrained**	0.07161	0.18614	15

[a)] Inertia is variance

Table S6. SIMPER results.

Table S7. Spearman's correlations between selected operational variables and bacterial genera. Only significant correlations (adjusted p-values < 0.05) are indicated. The genera that do not have a significant correlation, according to the established parameters, were eliminated from the table. Stronger correlations |0.70| are marked with colored cells: blue for negative and red for positive values.
